# Factors Affecting Glycemic Control in Patients with Type 2 Diabetes in Kalasin Province, Thailand: An Analytical Cross-Sectional Study

**DOI:** 10.3390/healthcare12191916

**Published:** 2024-09-25

**Authors:** Patcharin Phuwilert, Santisith Khiewkhern, Teerasak Phajan, Kasama Wongprachum, Jindawan Wibuloutai, Chitkamon Srichomphoo, Le Ke Nghiep, Kukiat Tudpor

**Affiliations:** 1 Faculty of Public Health, Mahasarakham University, Maha Sarakham 44150, Thailand; 60011460008@msu.ac.th (P.P.); kasama.w@msu.ac.th (K.W.); jindawan.w@msu.ac.th (J.W.); kukiat.t@msu.ac.th (K.T.); 2 Public Health and Environmental Policy in Southeast Asia Research Cluster (PHEP-SEA), Mahasarakham University, Maha Sarakham 44150, Thailand; 3 Department of Community Public Health, Sirindhorn College of Public Health Khon Kaen, Khon Kaen 40000, Thailand; tphajan@gmail.com; 4 Faculty of Health and Sports Science, Thaksin University, Phatthalung Campus, Phatthalung 93210, Thailand; rrangtime@gmail.com; 5 Vinh Long Department of Health, Vinh Long 85000, Vietnam; lekenghiep@gmail.com

**Keywords:** glycemic control, diabetic risk factors, health behavior, self-care behaviors, diabetes mellitus

## Abstract

**Background:** Optimal glycemic management is critical since it can predict all associated essential causes of death, even after accounting for other risk factors. **Objectives:** This study investigated the factors associated with glycemic control in patients with type 2 diabetes mellitus (T2DM), particularly self-care behaviors. **Methods:** An analytical cross-sectional study examined 385 patients with T2DM in 1 general and 17 community hospitals in Kalasin Province. The samples were collected using mixed-method sampling. Data were collected using a validated questionnaire with six parts and laboratory examination records between September 2021 and December 2022. Descriptive statistics (e.g., percentages and means ± SD) were used to analyze the data. The relationship between relevant factors and lack of glycemic control was analyzed by multivariate logistic regression using SPSS version 25. **Results:** Results showed that most patients were female (78.18%), and the mean age was 59.84 ± 9.05 years. Additionally, a significant proportion of the patients (79.74%) had poor glycemic control. Several factors are significantly associated with poor glycemic control in patients with T2DM. These factors included subjects under the age of 60 years (OR = 2.95, 95% CI: 1.57 to 5.54; *p*-value < 0.001), a diabetes duration of over 10 years (OR = 3.95, 95% CI: 1.90 to 8.22; *p*-value < 0.001), poor knowledge about diabetes (OR = 3.92, 95% CI: 1.59 to 9.67; *p*-value < 0.003), and inadequate self-care behaviors (OR = 6.12, 95% CI: 3.20 to 11.68; *p*-value < 0.001). **Conclusions:** In conclusion, proper interventions for controlling glycemic level behaviors are necessary to improve proper self-care behaviors in patients who have a low knowledge level of T2DM, have had diabetes for over ten years, and are aged < 60 years. This approach can reduce the likelihood of experiencing disabilities and economic hardship.

## 1. Introduction

Diabetes mellitus (DM) is a prevalent public health issue in Thailand and globally. Poor glycemic control may lead to various conditions, including blindness, kidney failure, heart attacks, stroke, and lower extremity amputation. Complications arising from inadequate glycemic control are chronic and result in high healthcare costs, adversely affecting the quality of life of individuals with diabetes and their families, as well as placing a burden on national healthcare expenditures. The World Health Organization (WHO) estimates that there are currently approximately 425 million diabetic patients worldwide, which is projected to reach approximately 629 million by 2045 [1]. According to the World Health Organization, diabetes mellitus was responsible for the deaths of 1.6 million individuals in 2016, making it the seventh leading cause of death [2]. In Thailand, the prevalence of T2DM is 9.00%, meaning that 2.5 million people, or around 10.00% of the country’s population, have the disease [3].

Maintaining appropriate glycemic control, mainly by monitoring glycated hemoglobin levels, is crucial because it can predict all related significant causes of death, even after considering other risk factors [4]. Patients with little or no glycemic control, especially those with T2DM, are at a higher risk of developing both acute and chronic complications [5]. Additionally, the lack of glycemic control in T2DM is a significant contributor to various conditions, such as blindness, kidney failure, heart attacks, stroke, and lower extremity amputations [6].

According to the Health Data Center system, patients in Thailand with T2DM often face significant challenges in maintaining good glycemic control. The system’s report shows that the percentage of T2DM patients with glycemic control was 26.7%, below the Ministry of Public Health of Thailand’s standard of ≥40% [7]. Several studies conducted in Thailand have found that demographic factors associated with glycemic control in Thai individuals include age, gender, BMI, knowledge, and healthcare behaviors [8,9,10]. Clinical factors related to glycemic control include HbA1c or fasting blood sugar levels, triglycerides, high-density lipoprotein (HDL), duration of diabetes, and follow-up appointments [8,9]. Regarding the social context, social support is linked to glycemic control [8].

Additionally, prior research conducted in other countries has identified numerous factors associated with glycemic control. These factors included physical activity, gender, age, duration of diabetes, body mass index, education, knowledge, self-care behaviors, and social support [11,12,13,14,15,16,17,18,19,20,21]. However, some studies did not find significant associations between gender, age, body mass index, duration of diabetes, education, and social support [22,23,24]. Adherence to strict self-care behaviors is crucial for managing diabetes and preventing severe morbidity and mortality. Such behaviors include making healthy food choices, engaging in physical activity, taking prescribed medications, and monitoring blood glucose levels [25]. However, self-care behaviors play a crucial role in controlling diabetes and related complications; conflicting findings in previous studies and the significance of this issue warrant further investigation [18]. Self-care sounds accessible to the general population, but it is a challenging issue among patients living in rural areas. Health literacy on T2DM is heterogeneous and geography-oriented [26]. Moreover, diabetic patients in rural areas received less diabetic health education than their urban counterparts [27]. Therefore, this study focused on self-care behaviors to identify the factors associated with poor glycemic control in patients with T2DM in rural areas of Kalasin, Thailand.

## 2. Materials and Methods

### 2.1. Research Design and Setting

This analytical cross-sectional study was conducted in the Kalasin Province, Thailand, from September 2021 to December 2022. Kalasin Province is in the northeastern region of Thailand and consists of rural and urban areas. As of 2022, 10,234 patients with T2DM are registered in the province’s 1 general and 17 community hospitals (district hospitals). The local population has specific dietary habits, such as high amounts of sticky rice, papaya salad with a high sugar content, and fermented foods, which may increase the risk of chronic diseases. The data collection process employed a stratified method, accounting for the prevalence of T2DM in each district of Kalasin Province.

### 2.2. Sample Recruitments

The sample comprised 385 adults aged ≥ 18 years with T2DM. These individuals were selected using a mixed methods approach, which involved the following steps: (1) stratifying the 18 community hospitals based on the calculated proportion of the sample assigned to each hospital, taking into account the population of T2DM patients and the study sample size; (2) applying a systematic sampling method, which accounted for the number of T2DM patients in each hospital relative to the study sample size; and (3) sampling from the lists of registered T2DM patients in the 18 community hospitals spread across 18 districts within Kalasin Province. This method was used to ensure a representative sample from diverse sources.

The sample size was determined based on a significance level of 0.05, an error of proportion estimate (e) of 0.05 [28], and a proportion of patients with uncontrolled blood sugar (*p*) of 0.56 [29], requiring 379 subjects. As such, 385 participants were included to cover a potential dropout rate of approximately 1.50%. The inclusion criteria were as follows: (1) patients with T2DM, both male and female, aged 18 years or older; (2) patients who had been registered and treated in hospitals in Kalasin for at least one year; (3) patients who had laboratory results from the last three fasting plasma glucose (FPG) tests or glycosylated hemoglobin (HbA1C) tests; (4) patients who participated voluntarily; and (5) verbal communication. The exclusion criteria were as follows: (1) patients with severe complications of the disease and (2) patients who could not provide information.

### 2.3. Data Collection

The principal investigator requested permission from the directors of the selected hospitals to use the lists of patients with T2DM at the diabetes clinic and collect data. Eighteen research assistants were trained by the principal investigator and administered the questionnaire to randomly selected T2DM patients. Each participant completed the questionnaire voluntarily, and the research assistant responded if the participants did not understand, which took approximately 25 min. The research assistants then checked the questionnaires for their completeness.

### 2.4. Research Instrument and Validation

The researchers developed a questionnaire in Thai with six different sections. These sections covered various aspects, including a checklist of sociodemographic characteristics with 6 items, a health status checklist with 11 items, a checklist to assess knowledge of DM and glycemic control (including 19 true and false items), a 3-point rating scale (agree, neutral, and disagree) to assess attitudes towards DM and glycemic control (with 17 items), a similar rating scale to measure self-care behavior (Regularly = practice 4 to 7 days/week, Sometimes = practice 1 to 3 days/week, and Never = no practice/week) (with 27 items), and another 3-level rating scale to examine social support (very satisfied, satisfied, and dissatisfied) (with 22 items). Each item had an item-objective congruence (IOC) index of greater than 0.50, as rated by five experts, including a physician, a nurse, and a health behavior expert, indicating a strong level of content validity for the questionnaires to ensure the content validity of the questionnaire. The questionnaire’s reliability was tested on patients with similar characteristics, using a sample of 30 patients with T2DM at Kantharawichai Hospital, a community hospital in Mahasarakham Province, similar to the hospital in the study. The reliability was assessed using the Kuder–Richardson 20 and Cronbach’s alpha. The Kuder–Richardson 20 coefficient of knowledge about DM and glycemic control was 0.73, while the Cronbach’s alpha coefficients for patients’ attitudes about DM, glycemic control, self-care behaviors, and social support were 0.72, 0.72, and 0.83, respectively.

### 2.5. Measurements and Interpretation

#### 2.5.1. Components of Demographic Characteristics

The demographic characteristics included age (in years), sex (male/female), body mass index (<23 kg/m^2^ or ≥23 kg/m^2^) [30], monthly income, education level (high school and below, undergraduate and above), knowledge about glycemic control and DM (good level, poor level), attitude about glycemic control and DM (good level, poor level), self-care behaviors (good level, poor level), and social support (good or poor).

Monthly income was calculated as the average household income per month, adjusted for sex and age, by computing the monthly equivalent income (monthly household income divided by the number of family members). Education level was categorized based on graduation status, with completion of high school or lower education considered “high school and below” and completion of an undergraduate or higher education as “undergraduate and above”.

#### 2.5.2. Patients with Type 2 Diabetes

The participants in this study were individuals diagnosed with T2DM based on the diagnostic criteria established by medical professionals and glycemic test results following the classification criteria set by the World Health Organization (WHO). Specifically, the criteria used were HbA1C levels ≥ 7% or FPG levels ≥ 126 mg/dL, assessed before the study and at least one year before enrollment. Medical doctors conducted the physical effort assessment following WHO routine guidelines.

#### 2.5.3. Glycemic Controlling

Glycemic control was evaluated in patients with T2DM who had been diagnosed for at least one year. The assessment was based on WHO criteria for classification using the last HbA1C test conducted before the study. The patients were categorized into two groups: The good glycemic control group consisted of patients with a previous glycemic test result of less than 7% or FPG levels below 126 mg/dL, while the uncontrolled blood sugar group included patients with a final glycemic test result of 7% or higher or FPG levels of 126 mg/dL or above, despite treatment.

#### 2.5.4. Knowledge About DM and Glycemic Control

In the knowledge section, the rating score for each item was assessed as correct = 1 point and incorrect = 0 points. The scores for each item are divided into three levels based on the percentage of the corrected score. A score of less than 60.00% was considered poor, between 60.01% and 79.99% was considered moderate, and a score of 80.00% and above was considered good. The total score was summed from the individual knowledge scores and categorized into two levels based on the mean score: a good level, defined as a score ≥ the mean, and a poor level, defined as a score below the mean.

#### 2.5.5. Attitude About DM and Glycemic Control

Each item in the attitude section was scored on three levels, namely agree = 3 points, neutral = 2 points, and disagree = 1 point for the positive questions, and agree = 1 point, neutral = 2 points, and disagree = 3 points for the negative questions. The division into three levels was based on the range, which was the highest score minus the lowest score, divided by the number of levels (3 − 1)/3 = 0.66. These levels were defined as follows: poor level (0.00 to 1.66), medium level (1.67 to 2.33), and good level (2.34 to 3.00). The total score of the attitude section was summed from the score of the individual items and categorized into two levels: a good level, defined as a score ≥ the mean, and a poor level, expressed as a score < the mean.

#### 2.5.6. Self-Care Behaviors

Individual ratings of self-care behavior for each item were scored as follows: regularly (4 days to all days per week) = 3 points, sometimes (1 day to 3 days/week) = 2 points, and never (has not been performed in a week but can sometimes be practiced in a month) = 1 point for the positive questions, and regularly = 1 point, sometimes = 2 points, and never = 3 points for the negative questions. Individual ratings of self-care behaviors were divided into three levels based on the range resulting from the highest score minus the lowest score divided by the number of levels, which equals (3 − 1)/3 = 0.66. These levels were defined as poor level (0.00 to 1.66), moderate level (1.67 to 2.33), and good level (2.34 to 3.00). The total score for self-care behavior was summed from the individual values of the individual items and divided into two levels based on the mean value: a good level (value ≥ mean value) and a poor level (value < mean value).

#### 2.5.7. Social Support

The individual scores for each item in the social support section were as follows: very satisfied = 3 points, satisfied = 2 points, and dissatisfied = 1 point for the positive questions, and very satisfied = 1 point, satisfied = 2 points, and dissatisfied = 3 points for the negative questions. The individual rating score for each item in the social support section was categorized into three levels based on the range: the highest score minus the lowest score, divided by the number of levels (3 − 1)/3 = 0.66. These levels were defined as follows: poor level (0.00 to 1.66), medium level (1.67 to 2.33), and good level (2.34 to 3.00). The total score for social support was summed from the individual values of the individual items and divided into two levels based on the mean value: a good level (value ≥ mean value) and a poor level (value < mean value).

### 2.6. Ethics Approval and Informed Consent

This study was approved by the Human Research Ethics Committee of Mahasarakham University (accreditation number 059/2019). It also received approval from Kalasin provincial public health officials (number KLS.REC 49/2564). It adhered to the Declaration of Helsinki’s ethical guidelines for human research 2013. All participants received the research objectives and essential information from the researchers. Written informed consent was obtained from each participant, who could decline participation at anytime. The researchers collected the data, which were then securely stored, locked, and password-protected to ensure the anonymity of the respondents. The results are presented only as an overview; no individuals have been identified. All methods used in this study were conducted according to relevant guidelines and regulations.

### 2.7. Data Analysis

The accuracy of the data was verified before entry, and analysis was performed using IBM SPSS (version 26.0) under the license of Mahasarakham University, Thailand. Descriptive statistics were used to summarize the demographic and health-related characteristics of the sample, including age, sex, body mass index, monthly income, education level, knowledge about DM, attitude about DM, self-care behaviors, and social support. Data were analyzed using frequency percentage and mean (SD) measures.

A bivariate analysis was performed using the independent *t*-test to compare the means of continuous data as the baseline for variables, such as age (in years), monthly income, duration of diabetes, BMI, knowledge score, attitude score, self-care behavior score, and social support score. Additionally, Chi-square tests or Fisher’s exact tests were used to compare the percentages of all variables, including demographic characteristics, age (<60 years/≥60 years) [31], sex (male/female), body mass index (<23 kg/m^2^ or ≥23 kg/m^2^) [30], monthly income (<2000/≥2000 Thai baht) (≈55 USD) based on the poverty line of the Ministry of Public Health of Thailand [32], education level (high school and below, undergraduate and above), knowledge about DM (sufficient level, insufficient level), attitude toward DM (good level, poor level), self-care behaviors (good level, poor level), and social support (good level, poor level).

Multivariate logistic regression analysis was conducted to identify the factors associated with poor glycemic control among patients with T2DM. The variance inflation factor (VIF) was used to check for collinearity of the dependent variables, and no evidence of collinearity was found, as all variables had a VIF value of less than 10. The Hosmer–Lemeshow test was performed to assess the goodness-of-fit of the model, and the Chi-square test showed no statistical significance (*p*-value of <0.05) (Chi-square = 10.25, *p*-value > 0.05), indicating that the data were reliable and that the model was appropriate for analysis. Based on a literature review, variables with a *p*-value < 0.25 in the bivariate analysis and those identified as potential factors related to poor glycemic control were included in the multivariate analysis. The adjusted odds ratios (adjusted ORs) and the 95% confidence intervals (CI) were calculated using multiple logistic regression with backward elimination. All statistical tests were two-tailed, and a significance level of 0.05 (*p*-value < 0.05) was used for data analysis.

## 3. Results

### 3.1. Demographic Characteristics

In total, 385 patients with T2DM were included in this study. Among the T2DM patients, 307 (79.74%) lacked glycemic control. T2DM patients, both male (79.76%) and female (79.73%), had similar percentages (*p*-value = 0.995). However, there was a significant difference in the mean age between the good glycemic control group (61.68 ± 9.51 years) and the uncontrolled blood sugar group (59.37 ± 8.89 years) (*p*-value = 0.044). In the uncontrolled blood sugar group, there was a higher proportion of individuals in the aged group < 60 years (85.13%) compared to the good glycemic control group (14.87%) (*p*-value = 0.007). No significant differences were observed between the two groups regarding education level, marital status, occupation type, monthly income, and duration of diabetes, all with a *p*-value < 0.05. When considering body mass index (BMI), a significant difference in the mean BMI was observed between the group with good glycemic control (23.82 ± 3.30) and the group with uncontrolled blood sugar (24.68 ± 3.21) (*p*-value = 0.035). Additionally, the proportion of individuals with BMI ≥ 23 kg/m^2^ in the uncontrolled blood sugar group (83.27%) was higher than that in the good glycemic control group (16.73%) (*p*-value = 0.006).

Moreover, T2DM patients showed a significant difference in their mean knowledge scores between the group with good glycemic control (17.00 ± 1.55) and the group with uncontrolled blood sugar (15.84 ± 2.36), with a *p*-value of 0.001. Additionally, the uncontrolled blood sugar group had a higher proportion of individuals with poor knowledge (92.31%) than the good glycemic control group (7.69%), with a *p*-value = 0.001). However, there was no significant difference in the mean attitude score between the group with good glycemic control (46.83 ± 3.03) and the group with uncontrolled blood sugar (46.41 ± 2.58) with a *p*-value of 0.211. Additionally, the proportions of individuals with poor and good attitudes were not significantly different between both groups, with a *p*-value greater than 0.05. The results indicated a significant difference in the mean self-care score between the group with good glycemic control (71.50 ± 4.97) and the group with uncontrolled blood sugar (66.53 ± 5.57), with a *p*-value of 0.001.

Furthermore, the uncontrolled blood sugar group exhibited a higher proportion of individuals with poor self-care behaviors (87.32%) than the good glycemic control group (12.68%), with a *p*-value = 0.001). However, there was no significant difference in the mean social support score between the group with good glycemic control (56.81 ± 6.07) and the group with uncontrolled blood sugar (55.54 ± 5.53), with a *p*-value of 0.078. Additionally, the proportion of individuals with poor and good social support was not significantly different between the groups, as indicated in Table 1, with a *p*-value greater than 0.05.

### 3.2. Complications Related to Diabetes

More than 50% of participants had diabetes-related complications, with most of them experiencing diabetic retinopathy and foot problems (11.69%), followed by diabetic foot complications (11.43%). Furthermore, the results confirmed that the group with a lack of glycemic control had more diabetes-related complications (60.59%) than the group with good glycemic control (30.77%). Specifically, the most common complications in the uncontrolled blood sugar group were diabetic retinopathy and foot problems (14.33%), followed by diabetic foot complications (13.68%), as shown in Table 1.

Alternative care encompasses any treatment or therapy outside mainstream medicine, including traditional or complementary medicine. While the majority of participants (91.68%) in this study did not use alternative care, the results indicated that the most frequently used form of alternative care was Reishi mushroom (3.90%), a Chinese herb (1.04%), as shown in Table 1.

### 3.3. Knowledge Levels on Diabetic Control

According to the findings, patients with type 2 diabetes had a satisfactory understanding of glycemic control and diabetes. Nonetheless, those belonging to the uncontrolled blood sugar group exhibited inadequate knowledge concerning the hereditary origins of diabetes (46.25%), as well as the fact that regulating blood glucose levels to the normal range can minimize the risk of disability and financial difficulties (48.21%). Please refer to Table 2 for further details.

### 3.4. Attitude Levels on Diabetic Control

The study results on the attitudes of patients with T2DM towards glycemic control and diabetes showed that the participants held a moderate attitude. The mean of the good glycemic control group was 2.29 ± 0.16, while that of the uncontrolled blood sugar group was 2.22 ± 0.16. However, they exhibited poor attitudes towards several aspects, such as believing that taking diabetic medication alone is sufficient to control blood sugar and that exercising is not necessary. The good glycemic control group’s mean was 1.27 ± 0.68, while that of the uncontrolled blood sugar group was 1.13 ± 0.46. Some participants also believed they could adjust their medication dosage independently to control their blood sugar levels. The mean glycemic control group was 1.38 ± 0.71, while that of the uncontrolled blood sugar group was 1.23 ± 0.57.

Additionally, diabetes can make one feel hopeless and helpless. Both groups were also found to have poor attitudes towards annual screening for diabetic nephropathy. The good glycemic control group’s mean was 1.26 ± 0.65, while the mean uncontrolled blood sugar group was 1.52 ± 0.85. Among the group with uncontrolled blood sugar levels, there was also a lack of attitude that maintaining good control requires regular medication use; the mean was 1.59 ± 0.90, and adherence to the doctor’s dietary recommendations (1.64 ± 0.91), which can be challenging, as shown in Table 3.

### 3.5. Self-Care Behaviors on Diabetic Control

The results regarding self-care behaviors indicated that both groups of participants exhibited deficiencies in several domains. Specifically, they demonstrated poor self-care behaviors, such as inadequate medication dosage adjustment. The mean of the good glycemic control group was 1.04 ± 0.17, while that of the uncontrolled blood sugar group was 1.08 ± 0.39. Furthermore, there were poor self-care behaviors related to the use of herbal remedies in conjunction with diabetes medication. The mean of the good glycemic control group was 1.09 ± 0.37, while that of the uncontrolled blood sugar group was 1.05 ± 0.24). Similarly, inadequate self-care behaviors were observed regarding the appropriate use of dietary supplements and diabetes medication. In the good glycemic control group, the mean was 1.10 ± 0.41, whereas in the uncontrolled blood sugar group, the mean was 1.11 ± 0.41. Additionally, the participants displayed poor self-care behaviors associated with the consumption of medications not prescribed by their doctors. The good glycemic control group’s mean was 1.08 ± 0.31; the uncontrolled blood sugar group’s was 1.08 ± 0.36. Moreover, both groups showed similar trends in limiting alcohol consumption. The good glycemic control group’s mean was 1.08 ± 0.31; the uncontrolled blood sugar group’s mean was 1.11 ± 0.34. Another noteworthy behavior was avoiding alcohol consumption during festivals. The mean of the good glycemic control group was 1.05 ± 0.22, whereas that of the uncontrolled blood sugar group was 1.11 ± 0.36. However, none of these results showed statistically significant differences when comparing the two groups, as shown in Table 4.

### 3.6. Social Support on Diabetic Control

The study results on social support among participants with T2DM revealed that, overall, both groups had a good level of social support. However, they exhibited poor social support in gaining knowledge about diabetes from the media. In the good glycemic control group, the mean was 1.64 ± 0.72, whereas in the uncontrolled blood sugar group, the mean was 1.61 ± 0.66. However, the two groups had no statistically significant difference, as indicated in Table 5.

### 3.7. Factors Related to Uncontrolled Glycemic Level in T2DM 

Bivariate analysis investigated the primary association between sociodemographic characteristics and uncontrolled blood sugar levels. The results showed that being in the age group < 60 years (*p*-value = 0.007), having a diabetes duration > ten years (*p*-value = 0.163), having a body mass index greater than 23 kg/m^2^ (*p*-value = 0.006), poor knowledge (*p*-value = 0.001), and inadequate self-care behaviors (*p*-value = 0.001) were associated with uncontrolled blood sugar among T2DM patients. These factors were included in the multivariate analysis, with a *p*-value < 0.25.

Age, diabetes duration, BMI, knowledge, and self-care behavior were independent variables. A lack of glycemic control was used as the dependent variable. Multivariate analysis was performed using binary logistic regression. The final model, determined using the backward elimination method, revealed that age, duration of diabetes, knowledge about DM, and self-care behaviors were significantly associated with a lack of glycemic control in patients with T2DM. Patients with T2DM younger than 60 were 3.20 times more likely to lack glycemic control than those over 60 (95% CI: 1.71 to 5.99; *p*-value = 0.001). Diabetes duration > 10 years increased the likelihood of lack of glycemic control by 3.95 times compared to those with ≤10 years (95% CI: 1.90 to 8.22; *p*-value < 0.001). Patients with poor knowledge were 3.92 times more likely to lack glycemic control than those with good knowledge (95% CI: 1.59 to 9.67; *p*-value = 0.003). Additionally, poor self-care behaviors were associated with a 6.12 times higher likelihood of lack of glycemic control compared to good self-care behaviors (95% CI: 3.20 to 11.68; *p*-value < 0.001), as shown in Table 6.

## 4. Discussion

Diabetes is a chronic disease with an increasing prevalence. The prevention and treatment of T2DM, as well as the management of its complications, are of paramount importance. These measures have primarily focused on glycemic control. However, owing to diverse cultures, geographical environments, socioeconomic conditions, dietary behaviors, exercise behaviors, and variations in healthcare services, there are distinct levels of glycemic control. This study explored the factors influencing the lack of glycemic control, specifically within Kalasin Province, Thailand.

This study showed that the prevalence of good glycemic control among T2DM patients was 20.26%, while the prevalence of lack of glycemic control was 79.74%. These findings were similar to the reported prevalence of good glycemic control in Anhui Province, China, at 22.97%) [33]. The study also found that the prevalence of lack of glycemic control in the female group (79.73%) was similar to that in the male group (79.76%). This result was inconsistent with a study in China that reported an association between women and glycemic control [33] and with research by Duarte et al., which reported that men had a stronger association with glycemic control [34]. These contradictory results may stem from variations in cultural and sociographic characteristics between this study and previous research. Previous studies have indicated associations between poor glycemic control and various factors, such as male sex, BMI, current smoking, and alcohol consumption [33].

Furthermore, a correlation study discovered a link between active smoking and an increased risk of poor glycemic control in men [35]. Regarding education, most participants fell into two categories: illiterate or had completed primary school (85.71%). This distribution was similar in both groups. Regarding marital status, the majority of participants were married (83.12%), and this proportion was also consistent across both groups. Additionally, a significant majority of participants identified as farmers (75.84%), which was similar in both groups. Furthermore, most participants reported having a monthly income of 2,000 Thai baht or more, consistent across both groups.

Participants in the uncontrolled blood sugar group had poor knowledge about the role of genetics as a cause of diabetes and the fact that maintaining blood sugar levels within the normal range can reduce the risk of disability and economic hardship. These results indicate that participants with poor glycemic control lacked a deep understanding of the serious consequences of poor blood sugar management [36]. Many held misconceptions, such as thinking that if they are taking diabetic medication, it is not necessary to control their diet or exercise. Additionally, some patients believed they could adjust their medication dosage to control their blood sugar levels. Others expressed feelings of hopelessness and helplessness due to their condition, and there was a recognition of the need for annual screening for diabetic nephropathy. These results indicate that patients with poor glycemic control rely more on medication than on other methods.

Regarding self-care behaviors in diabetes control, the participants demonstrated poor practices, particularly in adjusting medication doses correctly, taking herbal remedies alongside diabetes medication, using dietary supplements with diabetes medication, and taking medicines beyond what was prescribed. These behaviors were inconsistent with their attitudes. Additionally, the results suggest that self-care behaviors were not solely dependent on knowledge or attitude. The study results on social factors revealed that both groups exhibited poor social support in gaining knowledge about diabetes from the media. This finding was primarily because most participants were older adults who felt hopeless and helpless due to their conditions, which may have made them uncomfortable accessing information from the media [37].

This study identified four variables that appeared to be associated with a lack of glycemic control for T2DM. These included age, duration of diabetes, knowledge of diabetes mellitus, and self-care behaviors. A statistically significant association was found between age and glycemic control in patients with T2DM. Patients under the age of 60 years are more likely to experience poor glycemic control than those aged 60 years or older. This phenomenon can be attributed to the infrequent manifestation of symptoms, resulting in a lack of awareness of the severity and progression of the disease. Additionally, younger patients may exhibit lower self-management behaviors than their older counterparts [34,38]. These findings align with a previous study indicating that younger patients are at a heightened risk of elevated HbA1c levels [16,20] and exhibit poorer glycemic control than older patients [17,19,21].

Diabetes duration exceeding ten years was found to be associated with a lack of glycemic control in individuals with T2DM. This association can be attributed to the failure of beta cells, which results in a sustained lack of insulin secretion over time [39]. This lack of insulin secretion affects responsiveness to dietary interventions alone or with oral agents, making them less likely to be effective [40]. These findings align with other studies that have demonstrated a correlation between an extended duration of diabetes and poor glycemic control [5,13]. On the other hand, patients with a prolonged duration of diabetes might change their behavior, like during the COVID-19 lockdown, such as developing poor eating and exercise habits, becoming bored with their glycemic control routine, or receiving inadequate support from their family, which may influence their ability to control their blood sugar levels [41].

The findings of this study revealed an association between poor knowledge and poor glycemic control in individuals with T2DM. Patients with T2DM who possess inadequate knowledge are 3.92 times more likely to experience a lack of glycemic control compared to those with good knowledge. This discrepancy arises because poor knowledge makes patients unable to care for themselves adequately [14]. Conversely, patients with a good understanding of DM are better equipped to adhere to comprehensive care plans [14]. These findings are consistent with other studies indicating a significant association between knowledge about DM and glycemic control [42,43] and highlighting the connection between cognitive impairment and poor control [11].

Poor self-care behaviors are significantly associated with poor glycemic control in individuals with T2DM. Patients exhibiting poor self-care behaviors were 6.12 times more likely to experience poor glycemic control than those demonstrating good self-care behaviors. This association arises from the fact that patients with poor self-care behaviors tend to neglect self-care and lack interest in adopting healthy behaviors, particularly in terms of smoking and alcohol consumption. These findings are consistent with those of previous studies that highlighted the link between smoking, alcohol consumption, and lower levels of glycemic control [33]. Patients with greater confidence in managing self-care behaviors tend to exhibit lower HbA1c levels [14]. This observation aligns with another study that reported a connection between self-care management behaviors and poor glycemic control [44].

The current study identified three primary issues related to poor behavior: dietary control, physical activity, and stress management. These factors concur with prior research indicating that unhealthy eating habits are linked to inadequate glycemic control [11,15]. Patients with T2DM are at a higher risk of poorly controlled diabetes if they engage in insufficient physical exercise [5,13]. Moreover, stress significantly correlates with poorer glycemic control because stressed individuals are more likely to adopt unhealthy lifestyles [14,45].

Even though we employed effective data analysis methods, including bivariate and multivariate analyses, to examine the risk of various traits, factors, knowledge levels, attitudes, and self-care behaviors related to glycemic control among patients with T2DM, this study had some limitations. First, it was conducted exclusively in the northeastern region of Thailand, potentially restricting the applicability of the findings to other areas with varying cultural and demographic attributes. Secondly, self-reported questionnaires may have limited the acquisition of comprehensive information. Additionally, the study adopted a cross-sectional design, preventing the establishment of causal relationships based on the observed data and rendering the findings provisional. Inferences cannot be made from the observed relationships, thus making the findings temporary. However, this study provides valuable insights into the fundamental causes of poor glycemic control in patients with T2DM, which can be used to develop appropriate patient care approaches. Further research is needed to define or create a care model for T2DM patients with poor glycemic control. However, this analytical cross-sectional study could only explain the factors influencing self-care behavior.

## 5. Strengths and Limitations of This Study

This study involved a unique population living in rural areas in Thailand. This minor group of patients had relatively limited access to medical services. This present study investigated factors affecting glycemic control in this minor population. Our investigation of these factors paves the way for a community-oriented treatment plan. In the next phase of the study, we can make a comprehensive plan for these patients. We utilized a questionnaire with reasonable validity and internal consistency. The sample size was determined through variance calculation and multi-stage sampling to ensure a representative sample from all 18 subdistricts in Kalasin Province, Thailand. As a result, the findings could apply to patients with T2DM and may help predict glycemic control in this population. Effective data analysis methods, including bivariate and multivariate analyses, assessed the risks associated with various characteristics, knowledge levels, attitudes, and self-care behaviors related to glycemic control. The consistency of the results was supported by examining all risk factors through bivariate analysis and identifying significant factors in multivariate analysis. The outcomes of this study will be used to create a novel and tailored strategic plan for a specific population group. 

There are 4 levels of the 905 hospitals in the Thai healthcare system under the Ministry of Public Health: 35 regional (tertiary level, >500 beds covering 4–5 provinces), 96 general (secondary level, >200–500 beds covering a large district), and 774 community (primary, 30–200 beds covering a small district). Kalasin Province has a population of about one million inhabitants [46]. Given the approximately 10% prevalence of T2DM, there should be around 100,000 patients [47]. The province has 1 general hospital and 17 community hospitals. These hospitals provide primary healthcare for all patients, including 10,234 T2DM patients, presumably 10% of all T2DM patients available in the province. The rest of the patients have the right to consult physicians in tertiary hospitals or can be referred there when they have severe complications, like chronic diabetic nephropathy or chronic diabetic foot ulcers. Additionally, many with stable glycemic control receive healthcare services at smaller-size subdistrict health-promoting hospitals (under the Ministry of the Interior)—moreover, many self-pay for private hospitals. For this reason, another 90% might receive healthcare services outside the 18 hospitals examined in this study. This limitation might explain why we had a relatively small sample size.

## 6. Conclusions

In summary, poor glycemic control in T2DM patients was associated with younger age, a longer length of diagnosis, incorrect knowledge, and inadequate self-care behaviors. Therefore, this study can be used as a guideline for developing a care model for patients who lack glycemic control, with an emphasis on factors that are associated with a lack of glycemic control in T2DM patients, to prevent and reduce the complications of T2DM patients, including increased knowledge of the hereditary origins of diabetes, as well as the fact that regulating blood glucose levels in the normal range can minimize the risk of disability and financial difficulties. A better attitude is needed, such as not believing that taking diabetic medication alone is sufficient to control blood sugar and that exercising is not necessary. Experiencing diabetes can make one feel hopeless and helpless. Guidelines should also improve self-care behaviors on how to adjust medication dosages properly, the appropriate use of dietary supplements with diabetes medication, avoiding taking unprescribed medications, and refraining from smoking and drinking alcohol and sugary beverages.

## Figures and Tables

**Table 1:** The uncontrolled blood sugar of type 2 diabetes patients with sociodemographic characteristics (*n* = 385).

Characteristics	Good Glycemic Control (*n* = 78) *n* (%)	Uncontrolled Blood Sugar (*n* = 307) *n* (%)	*p*-Value (Exact Test, *t*-Test)
**Gender**			
Male	17 (20.24)	67 (79.76)	0.995
Female	61 (20.27)	240 (79.73)	
**Age** (years) (mean ± SD)	61.68 ± 9.51	59.37 ± 8.89	0.044 *
<60 years	29 (14.87)	166 (85.13)	0.007 *
≥60 years	49 (25.79)	141 (74.21)	
**Education**			
Illiterate/primary school	65 (19.70)	265 (80.30)	0.501
Secondary school/higher	13 (23.64)	42 (76.36)	
**Marital status**			
Married	62 (19.38)	258 (80.62)	0.338
Single, divorced, widowed	16 (24.62)	49 (75.38)	
**Occupation**			
Farmer	60 (20.55)	232 (79.45)	0.803
Other work	18 (21.21)	75 (78.79)	
**Income per month** (Baht) (mean ± SD)	3765.4 ± 4957.3	3638.0 ± 5890.3	0.865
<2000 (<55 USD)	26 (19.12)	110 (80.88)	0.680
≥2000 (≥55 USD)	52 (20.88)	197 (79.12)	
**Duration of diabetes** (years) (mean ± SD)	7.62 ± 5.70	9.19 ± 6.49)	0.051
≤10 years	37 (23.72)	119 (76.28)	0.163
>10 years	41 (17.90)	188 (82.10)	
**Body mass index** (kg/m^2^) (mean ± SD)	23.82 ± 3.30	24.68 ± 3.21	0.035 *
<23 kg/m^2^	32 (29.09)	78 (70.91)	0.006 *
≥23 kg/m^2^	46 (16.73)	229 (83.27)	
**Knowledge** (mean ± SD)	17.00 ± 1.55	15.84 ± 2.36	0.000 **
Poor	10 (7.69)	120 (92.31)	0.001 *
Good	68 (26.67)	187 (73.33)	
**Attitude** (mean ± SD)	46.83 ± 3.03	46.41 ± 2.58	0.211
Poor	32 (17.88)	147 (82.12)	0.278
Good	46 (22.33)	160 (77.67)	
**Self-care behaviors** (mean ± SD)	71.50 ± 4.97	66.53 ± 5.57	0.000 **
Poor	35 (12.68)	241(87.32)	0.001 *
Good	43 (39.45)	66 (60.55)	
**Social support** (mean ± SD)	56.81 ± 6.07	55..54 ± 5.53	0.078
Poor	25 (17.72)	134 (84.28)	0.063
Good	53 (23.45)	173 (76.55)	
**Diabetes-related complications**			
No	54 (69.23)	121 (39.41)	0.001 *
Yes	24 (30.77)	186 (60.59)	
Diabetic nephropathy	5 (6.41)	25 (8.14)	
Diabetic retinopathy	5 (6.41)	24 (7.82)	
Diabetic foot	2 (2.56)	42 (13.68)	
Cardiovascular diseases	1 (1.28)	0 (0.00)	
Diabetic nephropathy and retinopathy	5 (6.41)	9 (2.93)	
Diabetic nephropathy and foot	3 (3.85)	9 (2.93)	
Diabetic nephropathy and cardiovascular diseases	0 (0.00)	0 (0.00)	
Diabetic retinopathy and foot	1 (1.28)	44 (14.33)	
Diabetic retinopathy and cardiovascular diseases	1 (1.28)	0 (0.00)	
Diabetic nephropathy, retinopathy, and foot	1 (1.28)	33 (10.75)	
**Alternative care**			0.001 **
No	69 (88.46)	284 (92.51)	
Yes	9 (28.13)	23(71.87)	
Reishi mushroom (Chinese herb)	5 (6.41)	10 (3.26)	
Cordyceps (Chinese herb)	0 (0.00)	4 (1.30)	
Korean Ginseng	0 (0.00)	1 (0.33)	
Nan Chao Wei (Chinese herb)	1 (1.28)	3 (0.97)	
Garlic	1 (1.28)	0 (0.00)	
Other herbs	2 (2.57)	5 (1.63)	

* *p*-value < 0.05, ** *p*-value < 0.001.

**Table 2:** The correct knowledge level in each item of type 2 diabetes patients (*n* = 385).

The Issues of the Question	Good Glycemic Control (*n* = 78)		Uncontrolled Blood Sugar (*n* = 307)	
	*n* (%)	Interpretation	*n* (%)	Interpretation
1. Genetics is one of the causes of diabetes.	50 (64.10)	moderate	142 (46.25)	poor
2. Obesity causes diabetes.	57 (73.08)	moderate	190 (61.89)	moderate
3. Diabetic patients can eat without restrictions.	69 (88.46)	good	259 (84.36)	good
4. Diabetic patients can eat unlimited amounts of non-sweet fruits, such as guava and rose apple.	61 (78.20)	moderate	228 (74.27)	moderate
5. Diabetes cannot be cured completely.	71 (97.43)	good	298 (97.07)	good
6. If a diabetic patient forgets to take their medication, they can take double the amount the next time.	76 (97.43)	good	298 (97.07)	good
7. Diabetic patients should exercise at least five times a week for a minimum of 30 min per session.	69 (88.46)	good	298 (97.07)	good
8. Foods that cause high blood sugar levels are starchy foods, especially sticky rice.	72 (92.31)	good	290 (94.46)	good
9. The blood sugar level of diabetic patients should be between 80-130 mg/dl before meals.	67 (85.89)	good	228 (74.27)	moderate
10. Symptoms of diabetes include frequent and excessive urination, increased hunger, thirst, and weight loss.	69 (88.46)	good	295 (96.09)	good
11. Symptoms of low blood sugar include sweating, palpitations, weakness, irritability, lack of concentration, and dizziness.	73 (93.58)	good	298 (97.07)	good
12. High blood sugar can slow down the healing process of wounds.	54 (69.23)	moderate	190 (61.89)	moderate
13. Diabetic patients should receive an immediate evaluation for any complications as soon as they are diagnosed with diabetes.	75 (96.15)	good	299 (97.39)	good
14. Diabetic patients with prolonged high blood sugar levels are at risk of developing complications in their feet.	75 (96.15)	good	298 (97.07)	good
15. Poor blood sugar control in diabetic patients can lead to the development of kidney failure.	73 (93.58)	good	253 (82.41)	good
16. Poor blood sugar control in diabetic patients can cause damage to the cornea in the eyes, leading to vision impairment.	74 (94.85)	good	301 (98.05)	good
17. Diabetic patients with poor blood sugar control can lose foot sensation.	74 (94.85)	good	301 (98.05)	good
18. Managing blood sugar levels within normal ranges can decrease the likelihood of experiencing complications associated with diabetes.	77 (98.71)	good	301 (98.05)	good
19. Regulating blood sugar levels within the normal range can lower the chances of experiencing disability and economic hardship.	59 (75.64)	moderate	148 (48.21)	poor
Total	87.38	good	83.38	good

**Table 3:** The attitude level about diabetes in each item among type 2 diabetes patients (*n* = 385).

Issues	Good Glycemic Control (*n* = 78)		Uncontrolled Blood Sugar (*n* = 307)	
	Mean (SD)	Interpretation	Mean (SD)	Interpretation
1. To maintain good control of blood sugar levels, one must take medication regularly.	2.11 (0.76)	moderate	1.59 (0.90)	poor
2. Regular exercise can help control blood sugar levels.	2.78 (0.62)	good	2.87 (0.48)	good
3. Long-term use of diabetes medication can cause kidney damage.	2.91 (0.41)	good	2.85 (0.54)	good
4. Following the doctor’s dietary recommendations can be challenging.	2.17 (0.97)	moderate	1.64 (0.91)	poor
5. Reducing the consumption of starchy foods and sweet fruits can help control blood sugar levels.	2.97 (0.23)	good	2.94 (0.32)	good
6. Diabetic patients should not consume sweet desserts frequently.	2.94 (0.32)	good	2.82 (0.58)	good
7. If taking diabetic medication, it is not necessary to control one’s diet.	1.27 (0.68)	poor	1.13 (0.46)	poor
8. If you take medication for diabetes, exercising may not be necessary.	1.09 (0.40)	poor	1.07 (0.33)	poor
9. Consuming herbs with a bitter taste can help lower blood sugar levels.	2.59 (0.65)	good	2.46 (0.71)	good
10. Patients can adjust their medication dosage for diabetes to control their blood sugar levels.	1.38 (0.71)	poor	1.23 (0.57)	poor
11. Regular blood tests help patients learn to control their blood sugar levels.	2.87 (0.49)	good	2.91 (0.41)	good
12. Experiencing diabetes can make one feel hopeless and helpless.	1.26 (0.65)	poor	1.52 (0.85)	poor
13. Having diabetes can cause blurry vision.	2.86 (0.48)	good	2.86 (0.43)	good
14. Poor blood sugar control can lead to diabetic retinopathy.	2.59 (0.78)	good	2.88 (0.42)	good
15. Poor blood sugar control can lead to diabetic nephropathy.	2.95 (0.32)	good	2.88 (0.31)	good
16. Screening for diabetic nephropathy needs to be performed annually.	1.45 (0.82)	poor	1.56 (0.88)	poor
17. Controlling blood sugar levels can help reduce the risk of complications from diabetes.	2.91 (0.31)	good	2.82 (0.42)	good
Total	2.29 (0.16)	moderate	2.22 (0.16)	moderate

**Table 4:** Self-care behaviors level in each item among type 2 diabetes patients (*n* = 385).

Issues	Good Glycemic Control (*n* = 78)		Uncontrolled Blood Sugar (*n* = 307)		*p*-Value (*t*-Test)
	Mean (SD)	Interpretation	Mean (SD)	Interpretation	
**Dietary control**	2.17 (0.24)	moderate	2.20 (0.24)	moderate	0.324
1. Consume sugary food.	1.90 (0.44)	moderate	1.96 (0.44)	moderate	0.282
2. Eating sweet desserts between meals.	1.68 (0.50)	moderate	2.07 (0.58)	moderate	0.001
3. Eat fried and stir-fried food.	1.69 (0.57)	moderate	1.94 (0.65)	moderate	0.002
4. Consume sweet fruits with low sugar content, such as guava.	2.47 (0.57)	good	2.25 (0.56)	moderate	0.002
5. Eat boiled vegetables and steamed vegetables.	2.83 (0.38)	good	2.52 (0.54)	good	0.001
6. Eating fish meat.	2.88 (0.32)	good	2.62 (0.51)	good	0.001
7. Drinking sugary beverages.	2.53 (0.77)	good	2.17 (0.82)	moderate	0.001
8. Control the amount of food for daily consumption.	2.46 (0.70)	good	2.42 (0.64)	good	0.629
**Exercise**	2.67 (0.53)	good	2.42 (0.64)	good	
9. The frequency and duration of exercise in one week.	2.59 (0.61)	good	2.00 (0.80)	moderate	0.001
10. Choose an appropriate method of exercise.	2.73 (0.53)	good	2.04 (0.81)	moderate	0.001
11. There are proper exercise steps, such as warming up the body and starting with light exercises.	2.69 (0.54)	good	2.05 (0.81)	moderate	0.001
**Taking medication**	2.67 (0.21)	good	2.39 (0.200	good	0.001
12. Taking medication as prescribed by a doctor.	2.96 (0.19)	good	2.97 (0.21)	good	0.702
13. The proper way to take medication in case of a missed dose.	2.86(0.47)	good	2.86 (0.43)	good	0.986
14. Taking medication 30 min after a meal.	2.88 (0.36)	good	2.89 (0.40)	good	0.840
15. The proper way to adjust the dose of medication.	1.04 (0.17)	poor	1.08 (0.39)	poor	0.377
16. An appointment with a doctor.	2.95 (0.27)	good	2.92 (0.31)	good	0.434
17. Continuity in taking diabetes medication.	2.69 (0.71)	good	2.85 (0.51)	good	0.023
18. Taking herbal remedies along with diabetes medication.	1.09 (0.37)	poor	1.05 (0.24)	poor	0.245
19. Taking dietary supplements along with diabetes medication.	1.10 (0.41)	poor	1.11 (0.41)	poor	0.847
20. Taking other medications beyond what the doctor prescribed.	1.08 (0.31)	poor	1.03 (0.20)	poor	0.082
**Stress management**	2.32 (0.45)	moderate	1.98 (0.55)	moderate	0.001
21. Treating stress by having sufficient rest and sleep.	2.44 (0.73)	good	2.06 (0.77)	moderate	0.001
22. Treating stress through exercise.	2.35 (0.79)	good	1.82 (0.76)	moderate	0.001
23. Treating stress through meditation and mindfulness practice.	2.21 (0.63)	moderate	2.05 (0.65)	moderate	0.051
**Risky behaviors**	1.93 (0.21)	moderate	1.93 (0.26)	moderate	1.000
24. Smoking	2.94(0.34)	good	2.92 (0.36)	good	0.756
25. Avoiding of being near smokers.	2.79 (0.49)	good	2.79 (0.45)	good	1.000
26. Regular drinking of alcohol.	2.92(0.31)	good	2.89 (0.34)	good	0.431
27. Drinking alcohol only during the festival.	2.95(0.22)	good	2.89(0.36)	good	0.140
**Total**	2.07 (0.14)	moderate	1.97(0.16)	moderate	0.001

**Table 5:** The social support level in each item among type 2 diabetes patients (*n* = 385).

Issues	Good Glycemic Control (*n* = 78)		Uncontrolled Blood Sugar (*n* = 307)		*p*-Value (*t*-Test)
	Mean (SD)	Interpretation	Mean (SD)	Interpretation	
**Emotional support**	2.61 (0.37)	good	2.54 (0.45)	good	0.205
1. Received inquiries about illness symptoms from family members.	2.58 (0.63)	good	2.60 (0.59)	good	0.792
2. Received medication management from family members.	2.10 (0.89)	moderate	2.45 (0.78)	good	0.001
3. Received advice on how to behave from family members.	2.67 (0.57)	good	2.50 (0.64)	good	0.055
4. Received encouragement from family members.	2.78 (0.50)	good	2.63 (0.54)	good	0.026
5. Received encouragement to engage in leisure activities from family members.	2.55 (0.70)	good	2.28 (0.80)	moderate	0.006
6. Received love and good relationships from family members.	2.90 (0.31)	good	2.77 (0.45)	good	0.016
7. Received illness inquiries from community members.	2.54 (0.53)	good	2.47 (0.61)	good	0.353
8. Received encouragement from community members	2.81 (0.43)	good	2.68 (0.48)	good	0.029
**Health checkup support**	2.86 (0.36)	good	2.87 (0.33)	good	0.814
9. Received blood sugar checkups by healthcare professionals every appointment.	2.95 (0.27)	good	2.91 (0.31)	good	0.297
10. Received medication follow-up from healthcare professionals.	2.82 (0.50)	good	2.85 (0.41)	good	0.582
11. Received evaluation and examination of diabetes complications from healthcare professionals.	2.81 (0.51)	good	2.86 (0.40)	good	0.353
**Health information support**	2.45 (0.39)	good	2.43 (0.34)	good	0.653
12. Received advice on health care behavior from family members.	2.72 (0.53)	good	2.47 (0.61)	good	0.001
13. Received advice on healthcare behavior from public health volunteers.	1.96 (0.84)	moderate	1.94 (0.82)	moderate	0.848
14. Received advice on healthcare behavior improvement from healthcare professionals.	2.77 (0.53)	good	2.81 (0.47)	good	0.513
15. Received advice on glycemic control behavior from healthcare professionals.	2.79 (0.49)	good	2.87 (0.39)	good	0.126
16. Received advice on preventing diabetes complications from healthcare professionals.	2.82 (0.45)	good	2.87 (0.39)	good	0.328
17. Gained knowledge about diabetes from the media in the community.	1.64 (0.72)	poor	1.61 (0.66)	poor	0.725
**Healthcare facility support**	2.56 (0.38)	good	2.57 (0.30)	good	0.804
18. Received a home visit by healthcare personnel.	1.73 (0.86)	moderate	1.74 (0.81)	moderate	0.923
19. Received sufficient medication and healthcare supplies.	2.99 (0.11)	good	2.97 (0.23)	good	0.456
20. Received a personal health record book from healthcare personnel.	2.90 (0.41)	good	2.97 (0.18)	good	0.024
21. Received a diabetes self-care guide from a public health service provider.	2.79 (0.59)	good	2.90 (0.40)	good	0.051
22. Experienced convenience in traveling for the scheduled medical appointment.	2.40 (0.89)	good	2.30 (0.92)	Good	0.388
**Total**	2.59 (0.29)	good	2.56 (0.27)	Good	0.388

**Table 6:** Bivariate and multivariate analysis of uncontrolled blood sugar among type 2 diabetes patients by unconditional logistic regression.

Factors	Good Glycemic Control (*n* = 78) *n* (%)	Uncontrolled Blood Sugar (*n* = 307) *n* (%)	Crude OR	*p*-Value	Adjusted OR	*p*-Value	95% CI
Age							
≥60 years	49 (62.82)	141 (45.93)	1		1		
<60 years	29 (37.18)	166 (54.07)	1.99	0.007 *	3.20	<0.001 **	1.71 to 5.99
Duration of diabetes							
≤10 years	37 (47.44)	119 (38.76)	1		1		
>10 years	41 (52.56)	188 (61.24)	1.43	0.163	3.95	<0.001 **	1.90 to 8.22
Knowledge of DM							
Good	68 (87.18)	187 (56.10)	1		1		
Poor	10 (12.82)	120 (23.64)	4.36	0.001 **	3.92	0.003 **	1.59 to 9.67
Self-care behaviors							
Good	43 (55.13)	66 (21.50)	1		1		
Poor	35 (44.87)	241 (78.50)	4.49	<0.001 **	6.12	<0.001 **	3.20 to 11.68

OR = odds ratio, * *p*-value < 0.05, ** *p*-value < 0.001.

## Data Availability

Data are available upon request.
